# Effect of Fragmented DNA From Plant Pathogens on the Protection Against Wilt and Root Rot of *Capsicum annuum* L. Plants

**DOI:** 10.3389/fpls.2020.581891

**Published:** 2021-01-12

**Authors:** Luz Maria Serrano-Jamaica, Emiliano Villordo-Pineda, Mario Martín González-Chavira, Ramón Gerardo Guevara-González, Gabriela Medina-Ramos

**Affiliations:** ^1^Molecular Plant Pathology Laboratory, Polytechnic University of Guanajuato, Cortazar, Mexico; ^2^Molecular Markers Laboratory, Bajío Experimental Field, National Institute for Forestry, Agriculture and Livestock Research, Celaya, Mexico; ^3^C.A Biosystems Engineering, Autonomous University of Querétaro, Santiago de Querétaro, Mexico

**Keywords:** Phytophthora capsici L., Fusarium oxysporum, Rhizoctonia solani, wilt, elicitors, Capsicum annuum

## Abstract

Chili pepper (*Capsicum annuum* L.) production is affected by wilt and root rot, the most devastating disease caused by the pathogen complex of oomycete *Phytophthora capsici* Leon and the fungi *Fusarium oxysporum* Schlecht and *Rhizoctonia solani* Kühn, infecting roots, stems, leaves, and fruits. Fungicides are currently inefficient against this disease and have a high environmental impact. The use of elicitors is a sustainable alternative for inducing resistance to wilting and root rot. DNA fragments of an organism’s own origin (conspecific or self-DNA) have shown the ability to inhibit growth and activate defense mechanisms in some plant species. In this investigation, the effect of the fragmented DNA mixture of *Phytophthora capsici* L., *Fusarium oxysporum* S., and *Rhizoctonia solani* K. on the protection against wilt and root rot of *Capsicum annuum* L. plants was evaluated. Changes in plant performance, phenolics, and flavonoids contents, as well as gene expression involved in the production of defense metabolites after the fragmented and unfragmented DNA mixture in three concentrations (20, 60, and 100 μg mL^–1^) in chili peppers, were studied. The results obtained showed a decrease in plant height in 60 and 100 μg mL^–1^ concentrations in absence of pathogens. Moreover, the treatment with fragmented DNA 100 μg mL^–1^ showed significant increase in the content of phenolic compounds and total flavonoids as well as gene expression associated to plant defense in comparison with control plants. Interestingly, foliar application of DNA fragments of the pathogen complex to a concentration of 100 μg mL^–1^ caused a 40% decrease in the mortality of infected plants with the pathogens at 30 days post-inoculation compared with control plants inoculated with the pathogen complex but not sprayed with DNA fragments. These results suggested a perspective for application of fragmented DNA of these pathogens at the agricultural level in crop protection strategies to cope with wilt and root rot in *Capsicum*.

## Introduction

Chili pepper (*Capsicum annuum* L.) belongs to the *Solanaceae* family; according to specialists, it is native to Mexico and one of the main vegetables worldwide (Mejía-Teniente et al., 2013). In Mexico the production of chili pepper in 2018 reached 3.29 million tons (SIAP, 2018). The *Capsicum* genus is made up of 22 species of which five have been domesticated: *Capsicum baccatum*, *Capsicum chinense* (habanero), *Capsicum pubescens* (apple or peron), *Capsicum frutescens*, and *C. annuum* (jalapeño, serrano, black chili, width, walkway, and tree). The latter species is considered the most important because it groups the greatest diversity of cultivated or wild chili peppers (Mejía-Teniente et al., 2019). The chemical composition of *Capsicum annuum* L. fruits has been extensively studied, managing to identify compounds such as capsaicinoids, fatty acids, anthocyanins, glucosides, carotenoids, organic acids, aldehydes, ketones, alcohols, ethers, and sulfur compounds (Villa-Ruano et al., 2019).

The profitability of the crop has been seriously threatened for several decades by the disease known as chili pepper wilt and root rot, which causes premature death of the plant and is one of the most important phytosanitary problems due to the level of devastation and its dispersion in all producing areas of the world (Morán-Bañuelos et al., 2010). It is caused by the pathogen complex of the oomycete *Phytophthora capsici* Leon and the fungi *Fusarium oxysporum* Schlecht and *Rhizoctonia solani* Kühn, infecting both roots, stems, leaves, and fruits (Kim et al., 1997). These pathogens can cause yield losses of up to 100% (García-Rodríguez et al., 2010). The manifestation of the first symptoms is the wilting of the leaves, preserving the color, and hanging from the petioles to the plant; at the root there is a soft, odorless rot, until it reaches its loss or detachment (González-Chavira et al., 2009). At the base of the stem there is a brown stain that as the disease progresses becomes black, causing tissue necrosis and external injuries such as sunken cancers that gradually strangle the stem; the stems are kept upright with the hanging leaves, the nuts, and wrinkled (González-Chavira et al., 2009; Uribe-Lorío et al., 2014).

Due to the etiology of the pathological complex, it is difficult to establish the specific epidemiological cycle of infection in the plant and, therefore, it is difficult to establish a phytosanitary management program; adding to this, the high levels of genetic variability of the pathogens responsible for the disease (Walker and Bosland, 1999; Montero-Tavera et al., 2013). Among the main strategies for the control of this disease, cultivation practices that ensure well-drained soils, crop rotation, soil solarization, and chemical control are recommended (Parra and Ristaino, 2001; Hausbeck and Lamour, 2004; Piccini et al., 2019). However, a certain degree of resistance to fungicides has been developed; thus, these chemicals cannot protect sensitive crops from resistant pathogens (Piccini et al., 2019). Due to this situation, new alternatives for control have been sought, highlighting the use of rhizospheric fungi antagonistic to *Fusarium* spp., *R. solani*, and *P. capsici* L. (Jinag et al., 2006; Ramos-Sandoval et al., 2010; Robles-Yerena et al., 2010), the use of rhizobacteria antagonistic to *P. capsici* (Sang et al., 2008; Sang and Kim, 2012), inoculation with rhizospheric mycorrhizal fungi (Ozgonen and Erkilic, 2007), as well as the combined use of biofumigation and antagonistic microorganisms (Wang et al., 2014) and crop rotation (Lamour and Hausbeck, 2003). Another alternative has been the combined use of compost and grafts in resistant patterns (Gilardi et al., 2013). Currently, there are no varieties of *C. annuum* L. with total resistance to this pathogenic complex and the strategy of applying fungicides is followed, although the use of germplasm with possible resistance to these pathogens has been attempted (Chew et al., 2008; Babu et al., 2011; Koc et al., 2011). Based on the aforementioned, to cope with plant diseases it is important to have a strategy that minimizes or eliminates possible side-effects on other organisms different to the targets, as well as to avoid environmental contamination and the induction of resistance in the organisms to be controlled. A possibility for crop protection from diseases complying to these latter features is the management of the plant immune system using stress factors in an adequate dose (eustressic dose) using a controlled elicitation strategy (Vázquez-Hernández et al., 2019). Controlled elicitation has been successfully used in chili pepper to cope with geminivirus diseases (Mejía-Teniente et al., 2019).

The plant immune response is triggered by the recognition of exogenous molecules called molecular patterns associated with pathogens or microbes (PAMP: Pathogen-Associated Molecular Pattern/MAMP: Microbe-Associated Molecular Pattern) through transmembrane receptor proteins called pattern recognition receptors (PRR), which are found on the surface of plant cells and are responsible for guiding immunity to microbial infection in all plant species (Chisholm et al., 2006; Jones and Dangl, 2006; Bittel and Robatzek, 2007; Boller and Felix, 2009; Postel and Kemmerling, 2009; Bhat and Ryu, 2016; Choi and Klessig, 2016; Ferrusquía-Jiménez et al., 2020). PRR stimulation activates immunity triggered by PAMP (PTI: Pattern Triggered Immunity) (Dodds and Rathjen, 2010). Beside PAMPs and MAMPs, the plant defense system can also recognize endogenous signal molecules derived from the plant called “damage-associated molecular patterns” (DAMP) inducing immune responses (Seong and Matzinger, 2004; Mackey and McFall, 2006; Boller and Felix, 2009). PRRs perceive DAMPs or PAMPs/MAMPs and play an important role in resistance to pathogens (Zipfel, 2014), triggering responses mediated by jasmonic acid (JA) and salicylic acid (SA), thus also presenting resistance to herbivores (Duran-Flores and Heil, 2014; Ross et al., 2014).

A novel and interesting approach to be tested in controlled elicitation strategies is the use of randomly fragmented nucleic acids as suggested by Mazzoleni et al., 2014. Studies with litter decomposition identified that a general occurrence of litter autotoxicity was related to the exposure to self-DNA fragments, displaying species-specific inhibitory effects on plants by reduction of conspecific root growth and seed germination without affecting heterospecifics (Mazzoleni et al., 2015a). Further, these effects were also probed in bacteria, fungi, algae, protozoa, and insects, thus seeming to be a general biological process (Mazzoleni et al., 2015b). Moreover, based on these findings, the use of fragmented self-DNA for biological control has been proposed (Mazzoleni et al., 2014). Since that discovery, relevant evidence of the immunogenic function of extracellular DNA (eDNA) as immunity activator has been reported in plants (Barbero et al., 2016; Duran-Flores and Heil, 2018; Vega-Muñoz et al., 2018; Kawasaki, 2019). Based on this idea, on one hand, plants could recognize exogenous DNA from pathogen microorganisms and activate immunity (i.e., DNA acting as PAMP) (Ferrusquía-Jiménez et al., 2020). On the other hand, fragments of nuclear or mitochondrial DNA molecules that appear in the extracellular or cytosolic compartment indicating cellular dysfunction or damage, including loss of integrity of nuclei, mitochondria, or whole cells, (i.e., DNA acting as DAMP) can also be recognized (Vega-Muñoz et al., 2018). Duran-Flores and Heil (2018) reported that the application of fragmented self-DNA, but not heterologous DNA, generated a stronger immune response than the application of DNA from other species.

There are several genes related to plant defense, among which are phenylalanine ammonium lyase (*pal*), chalcone synthase (*chs*), manganese superoxide dismutase (Mn-*sod*), peroxidases (*pox*), glucanases (*glu*), and chitinases (*chi*) (Fernández-Herrera et al., 2012; Sudisha et al., 2012; Villar-Luna et al., 2015). Phenylalanine ammonium lyase (PAL) and chalcone synthase (CHS) are regulatory enzymes for the synthesis of phenylpropanoids and flavonoids in different plant tissues, respectively. Higher gene expression and activity of both enzymes is strongly related to resistant genotypes in several plant species against several diseases (Richins et al., 2010; Mejía-Teniente et al., 2013, 2019; Chávez-Díaz and Zavaleta-Mejía, 2019). Additionally, in response to stress, plants usually increase gene expression and activity of enzymes producing reactive oxygen species (ROS) (as superoxide dismutases) and those scavenging them (peroxidases or catalases) as a modulating system to establish an adequate stress response (Rodríguez-Calzada et al., 2019). In the present work, the effect of the fragmented DNA mixture of the pathogenic complex including *Phytophthora capsici* L., *Fusarium oxysporum* S., and *Rhizoctonia solani* K. on the protection against wilt and root rot of *Capsicum annuum* L. plants was evaluated. The results are discussed in regard the potential application of this strategy in chili pepper protection against wilt and root rot disease.

## Materials and Methods

### Biological Material

*In vivo* tests were carried out with seedlings of jalapeño pepper (*Capsicum annuum* L.), Gladiator MSC 983 variety from the MARSEED Company seed house, a variety susceptible to chili pepper wilt and root rot. Seedlings 45 days post germination were used, with 10–12 true leaves. They were transplanted into 1-L capacity unicel cups; sterile Sunshine 3M was used as substrate in greenhouse conditions, and their acclimatization was 7 days after transplantation.

Pathogen complex used in this study were isolated from Guanajuato state in Central Mexico affecting chili pepper plants with wilt and root rot disease. The pathogens were identified as *Phytophthora capsici* L. (identified using the method reported by Mitchell and Kannwischer-Mitchell, 1995 and the PCR strategy for species as reported by Silvar et al., 2005), *Fusarium oxysporum* F. Sp. capsici (Velarde-Félix et al., 2018), and *Rhizoctonia solani* (anastomosis group GA4, Montero-Tavera et al., 2013), provided by the Molecular Markers Laboratory of the National Institute of Agricultural and Livestock Forest Research (INIFAP) Campo Experimental Bajío.

### Extraction of Genomic DNA From *Phytophthora capsici* L, *Fusarium oxysporum*, and *Rhizoctonia solani*

Five disks (8 mm diameter) of potato dextrose agar medium with mycelium of each pathogen were placed in potato dextrose broth medium acidified with lactic acid at pH 4.0. Growth of the pathogens was carried out using incubation at 28°C, 180 rpm for 4 days for *P. capsici* and *R. Solani*. In the case of *F. oxysporum*, it was grown in aeration at room temperature. The mycelium was subsequently filtered through a nylon blanket, allowed to dry to remove excess moisture, and frozen. DNA extraction was performed using the CTAB method (Doyle and Doyle, 1987). From the frozen mycelium, it was macerated in a mortar with a 1:2 ratio of extraction buffer (CTAB 2%, 50 mM tris–HCl pH 8.0, 1.4 M NaCl, and 10 mM EDTA). The concentration was verified in the spectrophotometer (Multiskan GO) at 260 and 280 nm, and DNA integrity was verified by horizontal electrophoresis in 1.5% agarose and SB 1X buffer at 100 V for 20 min stained with SYBR Safe (Invitrogen), and displayed on the photo documentation (MiniBIS Pro).

### Genomic DNA Fragmentation

DNA fragmentation was performed using the Sonics Vibra Cell VCX130 sonotrode with a pulse emission per second of 26 KHz at 10 W, with an amplitude of 50%, for 10 min. The fragments were visualized in a 1.5% agarose gel in SB 1X buffer and stained with Gel red (Biotium) to determine the range of fragmentation obtained.

### Application of DNA Fragments

A mixture of fragmented DNA of *Phytophthora capsici* L., *Fusarium oxysporum* S., and *Rhizoctonia solani* K. was applied once by direct spraying on the leaves in three different concentrations (20, 60, and 100 μg mL^–1^). Fifteen plants with 10–12 true leaves and 7 days of transplant were used for each treatment. A treatment with unfragmented DNA consisting only with the genomic DNA obtained with the aforementioned methodology and not sonicated in order to have high-weight genomic DNA was used as another experimental control in the work. Table 1 shows the distribution of treatments.

**TABLE 1:** Design of experiments for the application of DNA fragments to pathogens.

	DNA fragments of the pathogen complex (DNA_FCF_)							
	20 μg mL^–1^	60 μg mL^–1^	100 μg mL^–1^	20 μg mL^–1^	60 μg mL^–1^	100 μg mL^–1^		
**Pathogen complex**	DNA_FCF20_ + CF	DNA_FCF60_ + CF	DNA_FCF100_ + CF				P_CF_	
**Pathogen free**				DNA_FCF20_	DNA_FCF60_	DNA_FCF100_		P_h_

*Subscript nomenclature: FCF, mixture of DNA fragments from the pathogen complex; CF, pathogen complex; P_*CF*_, Control of plants infected with the pathogen complex; P_*h*_, Control healthy plants; 20, 60, and 100, concentration of the mixture of the DNA fragments of the pathogen complex given in μg mL**^–^**^1^. As controls, the same fungi DNAs were applied unfragmented in the same concentrations onto the plants to compare results.*

### Inoculation of Plants With the Pathogenic Complex *Phytophthora capsici* L., *Fusarium oxysporum*, and *Rhizoctonia solani*

After 24 h of the application of the mixture of DNA fragments, the inoculation with the pathogen complex (CF) was carried out, for which a spore suspension of *F. oxysporum* was prepared based on the methodologies described by Kim et al. (1997) and Fernández-Herrera et al. (2007). The fungus was seeded in Petri dishes with potato dextrose (PD) agar medium; once it covered the entire box, 20 mL of sterile distilled water were added, and then with a scalpel the mycelium was scraped to separate the conidia; it was filtered to retain the mycelium and only allow the passage of the conidia; the filtrate product was collected and adjusted to a concentration of 1 × 10^6^ spores, determining the concentration with a hemacytometer. From *P. capsici*, a suspension of 1 × 10^5^ zoospores was added. The production of zoospores consisted of placing a disk of mycelium in PD broth, and then incubated in the dark for 15 days at 25°C with shaking at 120 rpm. Then, the mycelium was separated and washed three times with sterile distilled water, and incubated in sterile distilled water with agitation for 5 days; finally the concentration was adjusted with a hemocytometer Ezziyyani et al. (2004). In the case of *R. solani*, it was performed according to the methodology described by Büttner et al. (2004) and Berdugo et al. (2010). In 250 mL of PDA medium, four 8-mm-diameter mycelial disks were placed, with constant agitation at 100 rpm and 21°C, under darkness for 4 days; the mycelium was extracted and homogenized in a mortar, and the suspension of 2 mg of mycelium per mL of sterile distilled water was adjusted. Finally a concentration of 20 mg per plant was placed. For inoculation, 1 mL of the spore suspensions of *P. capsici* (final concentration of 1 × 10^6^ zoospores) and *F. oxysporum* (final concentration of 1 × 10^5^ spores) was placed on the stem base (neck) of the plant, as well as 20 mg of collected mycelium of *R. solani* (suspended in 10 mL of sterile distilled water). Once the three pathogens were placed, the plant neck was covered with the substrate.

### Measurement of Plant Morphological Variables

The height of the plant was measured in centimeters at 7, 14, 22, and 30 days after the application of the mixture of DNA fragments, using a flexometer, measuring from the base to the apex of the plant. Thirty days after the application of the mixture of DNA fragments, the length (using a flexometer) and the weight of the roots of the chili plants were determined in the different treatments. The dry weight of the root of the different treatments was determined, drying the tissue of the plant using an oven at 60°C for a time of 24–48 h until it reached a constant weight. In all cases, three biological replicates were carried out in the study.

### Severity and Incidence of the Disease

Plants were evaluated at 0, 7, 14, 22, and 30 days post inoculation (dpi) and application of treatments, to determine the proportion of infected plants in the different treatments (incidence). To measure severity, a gradual scale from 0 to 9 was used, reported by Bosland and Lindsey (1991), which takes into account the coloration of the plant, the state of the leaves, signs of disease, or if the plant is dead. On this scale, the interaction phenotype was standardized in 9 levels, where 0 = no response, vigorous, healthy (as uninoculated control); 1 = slight root darkening, vigorous, healthy; 3 = brown roots, slight stunting, very small lesions on stems; 5 = brown roots, small lesions stems, lower leaves wilted, stunted plants; 7 = brown roots, large lesions on stems, girdling, whole plant wilted, and stunted; and 9 = death. Even numbers were used for intermediate responses. A disease index value of 2 or less was considered resistant, and a value greater than 2 was susceptible. In all cases, three biological replicates were carried out in the study.

### Determination of Phenols and Total Flavonoids

The determination of phenolic compounds for all the evaluated treatments was carried out at 0, 1, 5, 15, and 30 days post-application of the mixture of fragmented or unfragmented DNA, according to Mejía-Teniente et al. (2013). Briefly for the sample preparation, 50 mg of leaf tissue was collected, ground with liquid nitrogen and homogenized in 2.5 mL of absolute methanol, and protected from light, with constant stirring at 150 rpm, 20°C for 24 h. After this time, it was centrifuged for 10 min at 5000 rpm, and the supernatant was recovered and stored at −20°C in the dark. After extraction, the total phenol content was determined by the Folin-Ciocalteu method, adapted for use in microplates. The reaction mixture consisted of 20 μL of the extract, 230 μL of distilled water, and 125 μL of Folin-Ciocalteu 1N reagent; the sample was homogenized and left to stand for 5 min, and 625 μL of 20% NaCO_3_ was added. The mixture was homogenized and left to stand for 2 h in the dark. After the resting time, 250 μL were taken to place on the microplate, and then the absorbance at 760 nm was determined on the Thermo Scientific Multiskan GO spectrophotometer. The amount of total phenols was expressed in micrograms equivalents of gallic acid per gram of fresh weight. For the determination of total flavonoids, it was performed according to Iqbal et al. (2015). For this method, 125 μL of the extract was mixed with 25 μL of 10% aluminum chloride, 25 μL of 1M potassium acetate, 375 μL of 80% methanol, and 700 μL of distilled water. This mixture was homogenized and allowed to stand 30 min at room temperature. Subsequently, the absorbance at 415 nm was determined on the Thermo Scientific Multiskan GO spectrophotometer. The amount of total flavonoids was expressed in micrograms of quercetin equivalents per gram of fresh weight. Three biological replicates were carried out in the study for these measurements.

### Gene Expression Analysis

Gene expression analysis was performed using RT-qPCR of two molecular markers of phenylpropanoids and flavonoid biosynthesis (phenylalanine ammonium lyase, *pal*, GenBank accession number AF081215) and chalcone synthase, *chs* (GenBank accession number FJ705842.1), as well as a gene marker of oxidative stress response (mitochondrial manganese superoxide dismutase, Mn-*sod*, GenBank accession number AF036936.2). As a housekeeping gene control, beta-tubulin (β-*tub* GenBank accession number EF495259.1) was used. For the extraction of total RNA, the TRIzol^®^ Reagent method (Ambion, Life Technologies) was used, following the manufacturer’s methodology. Complementary DNA (cDNA) synthesis was performed using the Clontech PCR-Select cDNA subtraction package (BD Biosciences). For the amplification of these genes in the treated chili pepper plants, primers used were previously reported by Rodríguez-Calzada et al. (2019); 200 ng of cDNA was used to perform qPCR, using Maxima SYBR Green qPCR Master Mix Thermo Scientific, in the C100 Touch Thermal Cycler device, BIORAD CFX96 Real Time System brand. Amplification conditions for *pal* and β-*tub* were 5 min at 94°C, 40 cycles of 94°C for 1 min, 55°C for 1 min, and 65°C for 5 s; while for Mn-*sod* and *chs*, it was 5 min at 94°C, 40 cycles of 94°C for 1 min, 58°C for 1 min, and 65°C for 5 s. This analysis was performed on chili pepper plants treated at 0, 1, and 5 days post application of the DNA fragments. Three biological replicates were analyzed in this study for all treatments. Relative gene expression was determined using the ΔΔCt methodology (Rodríguez-Calzada et al., 2019).

### Statistical Analysis

A completely randomized block design was carried out to evaluate the effect of DNA fragments of the pathogen complex as biocontrol agents of phytopathogens and the activation of defense mechanisms of chili plants in different concentrations. For gene expression, a two-way analysis of variance (ANOVA) was performed (two-way ANOVA) and the differences between treatments will be performed using the Tukey test *p* = 0.05, using the GraphPad Prism 8.0.2 software. In all the measured variables in this study, three biological replicates were analyzed and used in the statistical analysis.

## Results

### Genomic DNA Fragmentation

**FIGURE 1:**
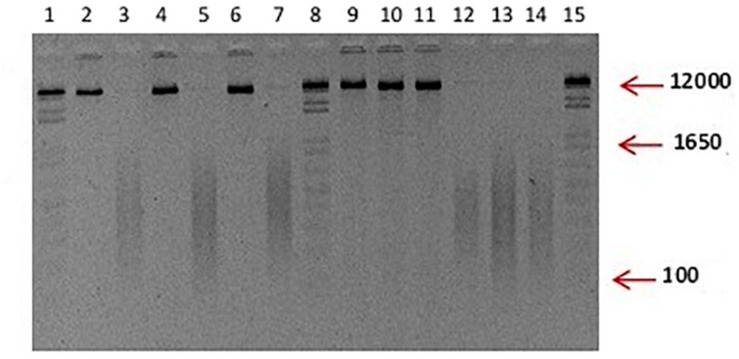
Typical visualization of unfragmented and fragmented DNA used in the study. Integral DNA of *P. capsici* and *F. oxysporum* in lanes 2, 4, 6, 9, 10, 11. Fragmented DNA is observed in lanes 3, 5, 7, 12, 13, and 14; Lane: 1, 8, and 15 100 ng of 1 Kb plus. Although not included, the DNA of *R. solani* used in the experiments displayed the same characteristics as the ones showed for *P. capsici* and *F. oxysporum*.

DNA fragment sizes were obtained from *P. Capsici* L., *F. oxysporum* S., and *R. Solani* K. in a size range of 100–1650 bp by direct sonication (Figure 1), which were applied in mixture to chili pepper plants.

### Effect of Mixture DNA Fragments Application on Plant Performance, Severity, and Incidence of “Wilt and Root Rot” Disease in Chili Pepper

**FIGURE 2:**
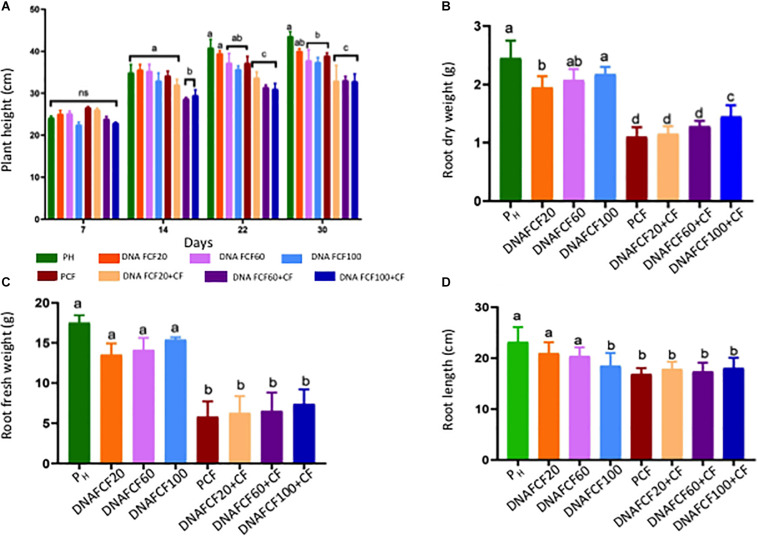
Morphological variables in chili pepper plants evaluated in the present study. **(A)** Plant height was evaluated in different times indicated in the figure and the rest of the variables (**B–D**, respectively, corresponding to dry weight, fresh weight, and length of roots) were evaluated at 30 dpi. Average ± standard deviation data of three biological replicates is shown. Two-way ANOVA statistical analysis, Tukey test *p* = 0.05. Equal lower case letters in each bar means no significant statistical difference for each analyzed variable. Symbology: FCF, mixture of DNA fragments from the pathogen complex; CF, pathogen complex; P_CF_, control plants infected with the pathogen complex; P_h_, control healthy plants; FCF 20, 60, and 100, different concentrations of the mixture of the DNA fragments of the pathogen complex given in μg mL^– 1^ evaluated in the study. The expression “ns” indicates no significant difference.

Plant height was determined at 7, 14, 22, and 30 days after DNA fragments application (dpa). As controls, unfragmented DNA of this pathogen complex was also evaluated in the study. The height of the plants treated with the mixture of DNA fragments of the pathogen complex (F_CF_) is presented in Figure 2A. At 7 dpa, there was no significant difference among the treatments. However, at 14 dpa, a significant decrease in height for the DNA_FCF__60_ + CF and DNA_FCF__100_ + CF treatments was observed (Figure 2A). Interestingly, at 22 and 30 dpa, a significant decrease in plant height was also observed in the treatment DNA_FCF__20_ + CF (Figure 2A).

**FIGURE 3:**
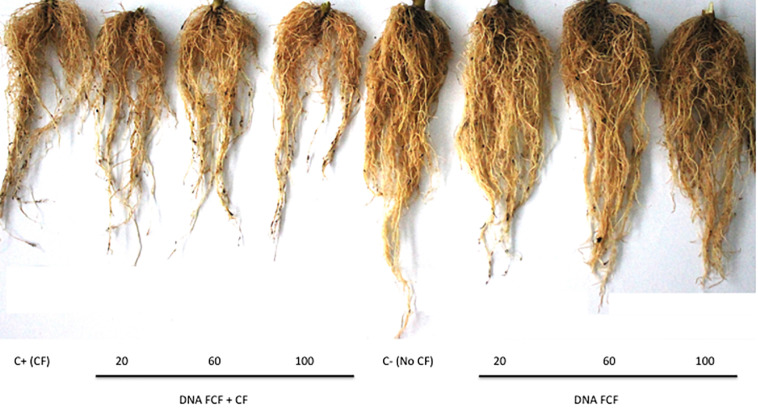
Root of plants infected with the pathogen complex at 30 dpi. From left to right are the Positive control (C+) pathogen complex control (P_CF_); (20), DNA_FCF__20_ + CF; (60), DNA_FCF__60_ + CF; (100), DNA_FCF__100_ + CF; (Ctrl-), P_h_; (E20) DNA_FCF__20_; (E60), DNA_FCF__60_, and (E100), DNA_FCF__100_. Symbology: FCF, mixture of DNA fragments from the pathogen complex; CF, pathogen complex; P_CF_, control plants infected with the pathogen complex; P_h_, control healthy plants; FCF 20, 60, and 100, different concentrations of the mixture of the DNA fragments of the pathogen complex given in μg mL^–1^ evaluated in the study.

The length and weight of the roots were also evaluated as another indicator of the disease in the chili pepper plants (Figures 2B–D, 3). Treatments with the mixture of DNA fragments of the pathogen complex (DNA_FCF__20_, DNA_FCF__60_, and DNA_FCF__100_) showed that in root length and weight the plants did not show a significant difference compared to the control healthy plants (P_h_) (Figures 2B–D, 3). However, significant decreases in length and weight of roots were displayed in the latter treatments when the pathogen complex was inoculated onto the plants (Figures 2B–D, 3).

**FIGURE 4:**
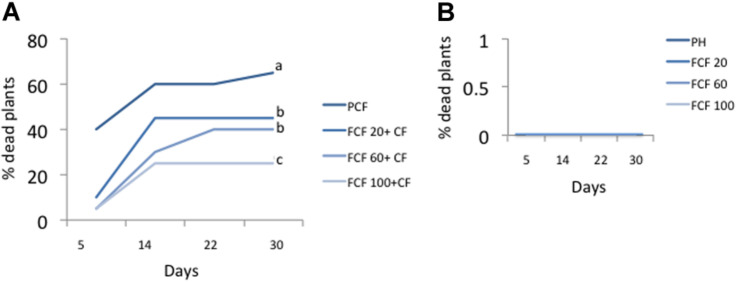
Percentage of dead plants at 5, 14, 22, and 30 days post-inoculation. **(A)** Percentage of dead plants in treatments inoculated with the pathogenic complex (CF) and treated with pathogens DNA (FCF) at 3 different concentrations. **(B)** Percentage of dead plants in healthy plant control (uninoculated with pathogenic control) and treated with pathogens DNA. Different letters in each line at day 30 in **(A)** indicates significant statistical difference according to Turkey test (*p* = 0.05). Simbology: FCF, mixture of DNA fragments from the pathogen complex; CF, pathogen complex; P_CF_, control plants infected with the pathogen complex; P_h_, Control healthy plants; 20, 60, and 100: concentrations of the mixture of the DNA fragments of the pathogen complex given in μg mL^–1^. The percentage of dead plants for the treatments with un-fragmented DNA had the same values as those shown in **(B)** (data not shown).

The symptoms of the disease were observable at 5 days post-inoculation (dpi) in control plants inoculated with the pathogen complex (disease control or P_CF_) (not shown). The percentage of dead plants at 30 days post-inoculation of the pathogen complex was 65% in disease controls (Figure 4A). A significant reduction in plant mortality at the same time was observed for the three fragmented DNA concentrations evaluated, showing 100 μg mL^–1^ as the best treatment causing a reduction of 40% in plant mortality in comparison with disease control (Figure 4A). Neither control healthy plants (P_h_) nor the treatments only with DNA (fragmented or not) caused plant mortality (Figure 4B).

**FIGURE 5:**
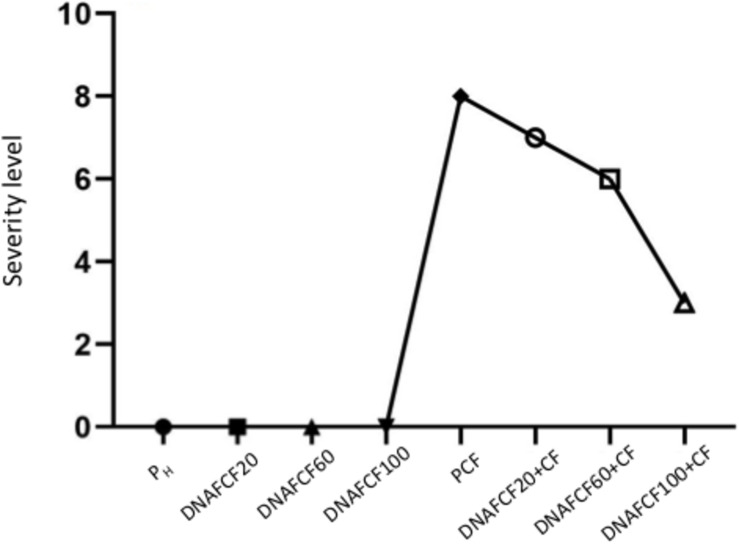
Maximum average severity level at 30 dpi. Simbology: FCF, mixture of DNA fragments from the pathogen complex; CF, pathogen complex; P_CF_, control plants infected with the pathogen complex; P_h_, control healthy plants; FCF 20, 60 and 100, different concentrations of the mixture of the DNA fragments of the pathogen complex given in μg mL^−1^ evaluated in the study.

**FIGURE 6:**
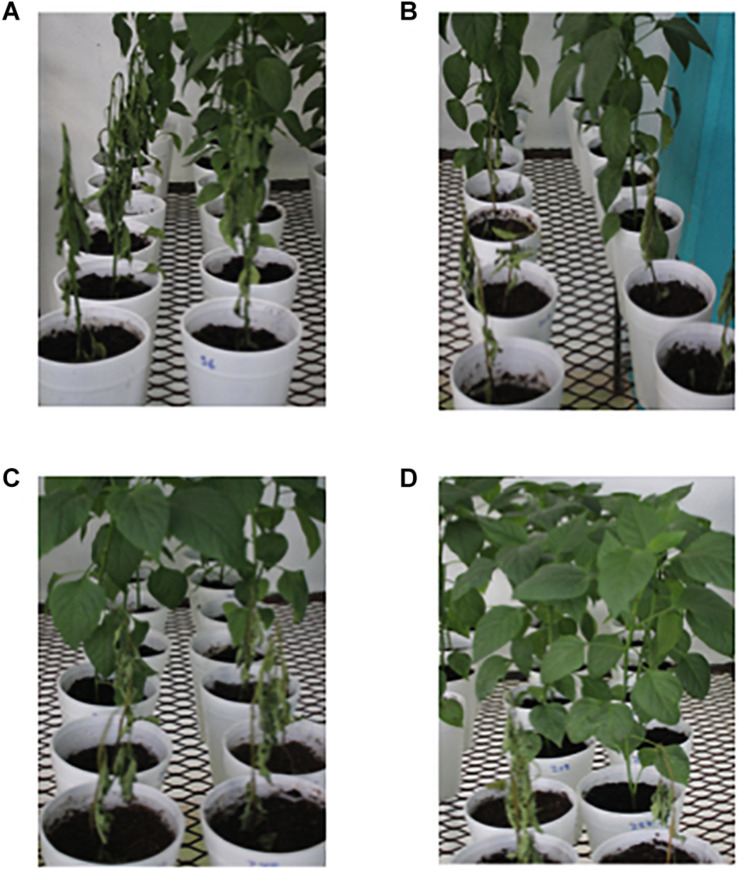
Plants treated with the pathogen complex. **(A)** Control plants plus pathogen complex (P_CF_) at 30 dpi. **(B)** Plants at 30 dpa of the DNA_FCF__20_ + CF treatment. **(C)** Plants at 30 dpa of the DNA_FCF__60_ + CF treatment. **(D)** Plants at 30 dpa of the DNA_FCF__100_ + CF treatment. Symbology: FCF, mixture of DNA fragments from the pathogen complex; CF, pathogen complex; P_CF_, control plants infected with the pathogen complex; P_h_, control healthy plants; FCF 20, 60, and 100, different concentrations of the mixture of the DNA fragments of the pathogen complex given in μg mL^– 1^ evaluated in the study.

In the case on non-dead plants, at 30 dpi a severity level of 8 was observed in disease control plants (P_CF_) (Figure 5). In the treatments inoculated with the pathogen complex and the mixture of fragmented DNAs of the three pathogens, the severity of disease at the same time was 7, 6, and 3 for the treatments with 20, 60, and 100 μg mL^–1^, respectively (Figure 5). Thus, the treatment 100 μg mL^–1^ caused a significant reduction in symptoms severity of 60%. Moreover, plants treated with unfragmented DNA of the pathogen complex and the controls without pathogen complex displayed no severity of the fungi disease as expected (Figure 5). A typical phenotype of wilt and root rot symptoms in the plants as well as resistant plants in each fragmented pathogen complex DNA treatments evaluated at 30 dpi is shown in Figure 6.

### Total Phenol and Flavonoid Levels in *C. annuum* Plants Treated With the Fragmented DNA of Pathogens

**FIGURE 7:**
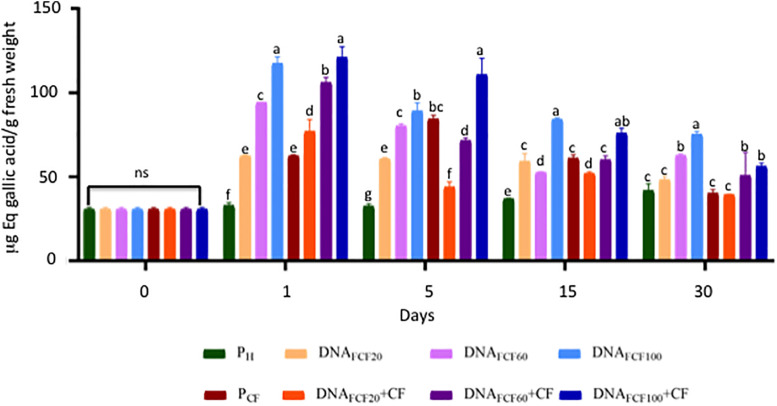
Total phenols (equivalents of gallic acid) of the plants treated with a mixture of DNA fragments of the pathogen complex at 0, 1, 5, 15, and 30 days post-DNA application. Average ± standard deviation data of three biological replicates is shown. Equal lower case letters in each bar for each time indicates significant statistical difference according to Tukey test (*p* = 0.05). Symbology: FCF, mixture of DNA fragments from the pathogen complex; CF, pathogen complex; P_CF_, control plants infected with the pathogen complex; P_h_, control healthy plants; FCF 20, 60, and 100, different concentrations of the mixture of the DNA fragments of the pathogen complex given in μg mL^– 1^ evaluated in the study. The expression “ns” indicates no significant difference.

**FIGURE 8:**
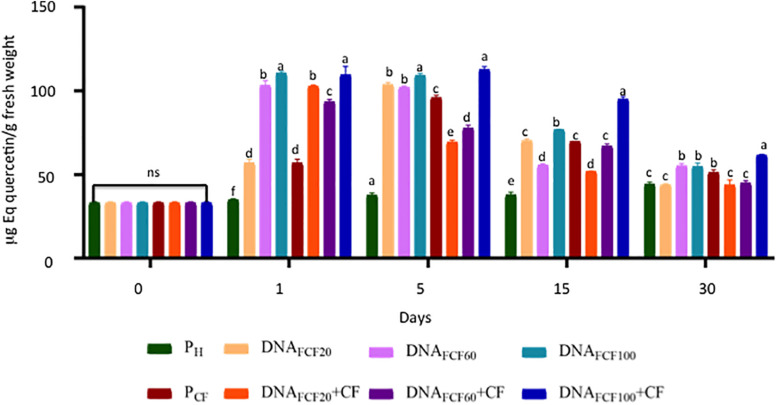
Total flavonoids (equivalents of quercetin) of plants treated with a mixture of DNA fragments of the pathogen complex at 0, 1, 5, 15, and 30 days post-DNA application. Average ± standard deviation data of three biological replicates is shown. Equal lower case letters in each bar for each time indicates significant statistical difference according to Tukey test (*p* = 0.05). Symbology: FCF, mixture of DNA fragments from the pathogen complex; CF, pathogen complex; P_CF_, control plants infected with the pathogen complex; P_h_, control healthy plants; FCF 20, 60, and 100, different concentrations of the mixture of the DNA fragments of the pathogen complex given in μg mL^– 1^ evaluated in the study. The expression “ns” indicates no significant difference.

A common defense mechanism displayed by plants to cope with the attack of pathogens is the production of phenols and flavonoids and the strength of cell membranes and walls (Dotor-Robayo and Cabezas-Gutierrez, 2014). Thus, the levels of phenols and flavonoids were measured in chili peppers treated with fragmented DNA of the pathogen complex (Figures 7, 8). As shown in Figure 7, all DNA fragment treatments as well as the pathogen complex significantly increased the total phenols in comparison with pH control plants from 1 to 30 dpa displaying Gaussian curve behavior. The DNA fragment concentration of 100 μg mL^–1^ increased the highest level of phenols in all times evaluated (Figure 7). In the case of flavonoids, the DNA fragments treatments displayed a similar behavior as in the one described for phenol levels throughout the evaluated times (Figure 8). Again the concentration of 100 μg mL^–1^ showed the highest increase in flavonoids (Figure 8).

### Gene Expression Levels

**FIGURE 9:**
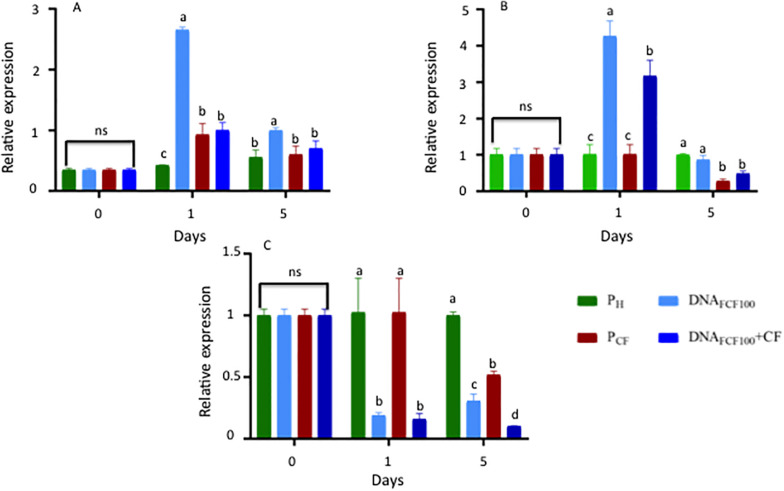
Relative gene expression of *pal*, *chs*, and Mn-*sod* at 0, 1, and 5 dpi in samples treated with a mixture of DNA fragments from the pathogen complex. **(A)** Relative expression of the phenylalanine ammonium lyase (*pal*). **(B)** Relative expression of the chalcone synthase (*chs*). **(C)** Relative expression of the Manganese Superoxide dismutase (Mn-*sod*). Average ± standard deviation data of three biological replicates is shown. Equal lower case letters in each bar for each time indicates significant statistical difference according to Tukey test (*p* = 0.05). Symbology: FCF, mixture of DNA fragments from the pathogen complex; CF, pathogen complex; P_CF_, control plants infected with the pathogen complex; P_h_, control healthy plants; FCF 20, 60, and 100, different concentrations of the mixture of the DNA fragments of the pathogen complex given in μg mL^– 1^ evaluated in the study. The expression “ns” indicates no significant difference.

The expression patterns of the *pal*, *chs*, and Mn-*sod* genes were analyzed at 0, 1, and 5 dpa in the treatments P_*S*_, P_CF_, DNA_FCF__100_, and DNA_FCF__100_ + CF (Figure 9). The time intervals evaluated were chosen based on previous studies in our group in chili pepper in response to other stress response factor (hydrogen peroxide) protecting chili peppers against geminivirus disease (Mejía-Teniente et al., 2019). The highest level of *pal* gene expression at 1 dpa was shown by the DNA_FCF__100_ treatment with significant difference compared with control healthy plant (Ph), control plants inoculated with the pathogen complex (P_CF_), and the DNA_FCF__100_ + CF treatment (Figure 9A). Comparing the treatments DNA_FCF__100_ + CF with control treatment P_CF_ no significant difference was shown. However, control of healthy plants (P_h_) displayed a significant difference (Figure 9A). At 5 dpa the gene expression level of *pal* decreases more than 50% for the DNAFCF100 treatment, but it presents a significant difference compared to the control of healthy plants (P_h_), the control of plants inoculated with the pathogen complex (P_CF_), and the DNA_FCF__100_ + CF treatment, with DNA_FCF__100_ treatment showing a higher level of expression. When comparing the DNA_FCF__100_ + CF treatment with the control Ph and the control P_CF_, there was no significant difference (Figure 9A). For *chs* expression, a higher level was observed at 1 dpa in the DNA_FCF__100_ treatment, showing a significant difference compared with the control P_h_, the control P_CF_, and the DNA_FCF__100_ + CF treatment (Figure 9B). Comparing treatment DNA_FCF__100_ + CF with control P_CF_ and the control P_h_, a significant difference was observed, showing the highest level of expression for DNA_FCF__100_ + CF treatment. At 5 dpa, the level of *chs* gene expression decreased for the DNA_FCF__100_ treatment, with no significant difference compared to the control P_h_, but showing significant difference when compared to the control P_CF_ and the DNA_FCF__100_ + CF treatment. When comparing the DNA_FCF__100_ + CF treatment with the control Ph, a significant decrease in the expression level was shown for the DNA_FCF__100_ + CF treatment. Comparing the DNA_FCF__100_ + CF treatment with the control P_CF_, no significant difference was shown (Figure 9B). Mn-*sod* did not show changes in the expression levels in control plants P_h_ and P_CF_ at 1 dpa, but it presented a significant difference compared with the DNA_FCF__100_ and DNA_FCF__100_ + CF treatments (Figure 9C). At 5 dpa, the DNA_FCF__100_ control showed an increase in expression level with respect to 1 dpa. The level of Mn-*sod* gene expression for DNA_FCF__100_ + CF decreased from 1 to 5 dpa (Figure 9C). In general, it was observed that Mn-*sod* showed a decrease in the expression levels in plants treated with the mixture of DNA fragments of the pathogen complex (Figure 9).

## Discussion

Recent studies have reported that extracellular self-DNA stimulates plant responses, activating the immune response such as PTI, by activating MAPKs, production of reactive oxygen species, depolarization of the membrane, activating ion flows of calcium, and thus providing resistance to plants against pathogens (Duran-Flores and Heil, 2014, 2018; Barbero et al., 2016; Hou et al., 2019). It has been reported that once the immune responses are activated in plants, there is an energy consumption affecting their growth, since several of the plant’s resources are used to defend itself of the disease (Walters and Heil, 2007; Yakushiji et al., 2009; Duran-Flores and Heil, 2018; Quintana-Rodríguez et al., 2018). In our work, there was a decrease in the height and root density (root length and weight) of the plants in the treatments inoculated with the pathogens alone or together with the DNA fragments of the pathogenic complex, possibly because their resources are being used to cope with the disease and generate resistance or disease management as suggested elsewhere (Bhat and Ryu, 2016). Recent advances in uncovering signal transduction networks have revealed that defense trade-offs are often the result of regulatory “decisions” by the plant, enabling it to fine-tune its phenotype in response to diverse environmental challenges (Züst and Agrawal, 2017).

In another study, Duran-Flores and Heil (2018), with the use of DNA fragments of *Phaseolus vulgaris*, significantly decreased infections caused by *Pseudomonas syringae* in *Phaseolus vulgaris*. This latter study is a case of using DNA as DAMP for inducing plant immunity. Our study is a case of DNA as PAMP with the same goal as the report of Duran-Flores and Heil (2018) in a different plant species (*Capsicum annuum* L.). With the application of the DNA fragments of the pathogen complex, a decrease of 40% was obtained in the control of wilt and root rot in chili peppers at a concentration of 100 μg mL^–1^. Our study also showed that the higher the concentration of fragmented DNA the higher the control of the disease at severity and dead plant levels. Both PAMPs and DAMPs have the ability to reprogram plants transcriptomically and metabolomically acting as amplifiers for PTI (Hou et al., 2019). The phenylpropanoid route is one of the main defense mechanisms. In this work, with the application of the DNA fragments from root rot phytopathogens onto chili plants, it was possible to increase the levels of phenolics and flavonoids. In recent studies it has been reported that with the exogenous application of hydrogen peroxide to protect chili peppers against geminivirus diseases, the protection level was directly related to increases in phenols and flavonoids levels (Mejía-Teniente et al., 2019). Moreover, the increase of the phenol and flavonoid levels displayed a Gaussian curve behavior during the time evaluated in the present study. Similar results were obtained in other works studying several elicitors as salicylic acid, chitosan, and hydrogen peroxide in chili peppers (Mejía-Teniente et al., 2013). In another study, Vega-Muñoz et al. (2018) reported that DNA fragments of the lettuce plant (*Lactuca sativa* L.) increased the concentrations of phenols and total flavonoids, activating defense responses to oxidative stress and the synthesis of secondary metabolites for stress management.

Koc et al. (2011) reported that *pal* activity increased when *P. capsici* infection occurs, becoming one of the first responses after infection, since it participates in the biosynthesis of phenolic compounds and phytoalexins. PAL is correlated with the defense in plants of *Capsicum annuum* against pathogens (Jung et al., 2004). In this work, with the application of the DNA fragments, a greater expression of *pal* and *chs* was achieved at 1 dpa was directly related to the increase in the concentrations of phenols and flavonoids of the samples analyzed as aforementioned. Chávez-Díaz and Zavaleta-Mejía, 2019 reported that the expression of *pal* in *Capsicum* plants, presenting an accumulation of phenols and phytotoxic compounds that activate the hypersensitive response and the synthesis of salicylic acid. Interestingly, the protection levels of chili pepper treated with fragmented DNA of the pathogenic complex at 100 μg mL^–1^ was correlated with higher levels of phenylpropanoids and gene expression associated to stress response in plants. Finally, our results also suggested that only one application of fragmented DNA of the pathogenic complex at 100 μg mL^–1^ onto juvenile chili peppers (10–12 true leaves) was enough to significantly diminish the wilt and root rot disease in 40%. Thus, fragmented DNA from pathogenic-origin used as PAMP in chili peppers might be an interesting sustainable strategy to be included in the management of this disease worldwide. Mazzoleni et al. (2014) suggested that self-DNA applications into soil might be a strategy to cope with soil phytopathogens. In our research, we use DNA from soil-living phytopathogens applied onto chili pepper plants (i.e., as PAMP) not into the soil having significant crop protection. It is plausible supposedly that a combined strategy in which an adequate dose of DNA from these pathogens used as PAMP (as in the present work) and applied into the soil to inhibit the growth of these phytopathogens (i.e., as DAMP) might increase the protection of chili peppers against wilt and root rot disease. These strategies are currently being tested in our group in experiments at open field and greenhouse levels, trying to provide a sustainable strategy to protect chili peppers against this important disease thus increasing plant growth and yields with minimal side-effects to nature.

## Conclusion

The mixture of DNA fragments of the three phytopathogens evaluated in this research applied in a concentration of 100 μg mL^–1^ induced the immune system of the chili plants and caused a control on the severity of the wilt of the chili caused by these phytopathogens, reducing mortality by 40%. In addition, a significant decrease of 60% in symptom severity compared to control plants inoculated with the pathogen complex was also obtained.

## Data Availability Statement

The raw data supporting the conclusions of this article will be made available by the authors, without undue reservation.

## Author Contributions

LS-J carried out all the experimentation as part of her Master in Science thesis. EV-P and MG-C carried out the design and advisory in phytopathological tests in the greenhouse. GM-R and RG-G conceived the research and participated in gene expression and biochemical tests carried out in the research, additionally both wrote the manuscript. All authors contributed with critical review of the manuscript before submission.

## Conflict of Interest

The authors declare that the research was conducted in the absence of any commercial or financial relationships that could be construed as a potential conflict of interest.
